# Prevalence of hepatitis D virus infection in sub-Saharan Africa: a systematic review and meta-analysis

**DOI:** 10.1016/S2214-109X(17)30298-X

**Published:** 2017-09-11

**Authors:** Alexander J Stockdale, Mas Chaponda, Apostolos Beloukas, Richard Odame Phillips, Philippa C Matthews, Athanasios Papadimitropoulos, Simon King, Laura Bonnett, Anna Maria Geretti

**Affiliations:** a Malawi-Liverpool-Wellcome Trust Clinical Research Programme, Blantyre, Malawi; b Institute of Infection and Global Health, University of Liverpool, Liverpool, UK; c Institute of Translational Medicine, University of Liverpool, Liverpool, UK; d Department of Biostatistics, University of Liverpool, Liverpool, UK; e Department of Medicine, Kwame Nkrumah University of Science and Technology, Kumasi, Ghana; f Department of Medicine, Komfo Anokye Teaching Hospital, Kumasi, Ghana; g University of Oxford and Oxford University Hospitals NHS Foundation Trust, Oxford, UK

## Abstract

**Background:**

Hepatitis D virus (also known as hepatitis delta virus) can establish a persistent infection in people with chronic hepatitis B, leading to accelerated progression of liver disease. In sub-Saharan Africa, where HBsAg prevalence is higher than 8%, hepatitis D virus might represent an important additive cause of chronic liver disease. We aimed to establish the prevalence of hepatitis D virus among HBsAg-positive populations in sub-Saharan Africa.

**Methods:**

We systematically reviewed studies of hepatitis D virus prevalence among HBsAg-positive populations in sub-Saharan Africa. We searched PubMed, Embase, and Scopus for papers published between Jan 1, 1995, and Aug 30, 2016, in which patient selection criteria and geographical setting were described. Search strings included sub-Saharan Africa, the countries therein, and permutations of hepatitis D virus. Cohort data were also added from HIV-positive populations in Malawi and Ghana. Populations undergoing assessment in liver disease clinics and those sampled from other populations (defined as general populations) were analysed. We did a meta-analysis with a DerSimonian-Laird random-effects model to calculate a pooled estimate of hepatitis D virus seroprevalence.

**Findings:**

Of 374 studies identified by our search, 30 were included in our study, only eight of which included detection of hepatitis D virus RNA among anti-hepatitis D virus seropositive participants. In west Africa, the pooled seroprevalence of hepatitis D virus was 7·33% (95% CI 3·55–12·20) in general populations and 9·57% (2·31–20·43) in liver-disease populations. In central Africa, seroprevalence was 25·64% (12·09–42·00) in general populations and 37·77% (12·13–67·54) in liver-disease populations. In east and southern Africa, seroprevalence was 0·05% (0·00–1·78) in general populations. The odds ratio for anti-hepatitis D virus detection among HBsAg-positive patients with liver fibrosis or hepatocellular carcinoma was 5·24 (95% CI 2·74–10·01; p<0·0001) relative to asymptomatic controls.

**Interpretation:**

Findings suggest localised clusters of hepatitis D virus endemicity across sub-Saharan Africa. Epidemiological data are needed from southern and east Africa, and from patients with established liver disease. Further studies should aim to define the reliability of hepatitis D virus testing methods, identify risk factors for transmission, and characterise the natural history of the infection in the region.

**Funding:**

Wellcome Trust, Royal Society.

## Introduction

Liver cirrhosis and hepatocellular carcinoma accounted for more than 3% of adult deaths in sub-Saharan Africa in 2013, and this proportion is rising.^1^ Chronic infection with hepatitis B virus, which has an estimated population prevalence of 8·8%, is the main cause.^2^ Hepatitis D virus (also known as hepatitis delta virus) is a small satellite sub-virus that requires the presence of HBsAg to propagate.^3^ Hepatitis B and D virus infections can occur simultaneously, which frequently results in clearance of both viruses, or as superinfection in chronic hepatitis B virus carriers, which often leads to persistence of hepatitis D virus.^4^ Individuals concomitantly infected with hepatitis B and D viruses can mount transient serological responses before clearance; thus, screening for hepatitis-D-virus-specific antibody reliably identifies cases of chronic super-infection but is less accurate for ascertainment of overall rates of exposure.^4^

Chronic hepatitis B and D virus co-infection is associated with expedited progression to cirrhosis, and has been characterised as the most severe form of viral hepatitis.^5^ In sub-Saharan Africa, where 4·8% of adults are living with HIV, hepatitis B virus infection and HIV often coexist, albeit with geographical variations.^6^, ^7^ Co-infection with HIV, hepatitis B virus, and hepatitis D virus could be an important specific cause of liver-related morbidity and mortality.^8^, ^9^ The only treatment for hepatitis D virus is pegylated interferon-α, and less than 30% attain sustained virological responses. Novel drug classes, including prenylation and entry inhibitors, are in development.^4^, ^10^, ^11^

Despite widely held assumptions of hyper-endemicity, no previous systematic analyses have been done of the epidemiology of hepatitis D virus in sub-Saharan Africa. Routine testing for hepatitis D virus is rare in clinical practice, and because of low rates of case ascertainment, the overall contribution of the virus to the burden of liver disease in the region is unknown.^5^ We aimed to define the available evidence on the prevalence of hepatitis D virus in sub-Saharan Africa and identify research needs.

**Research in context**

**Evidence before this study**

We searched PubMed, Embase, and Scopus with the terms “hepatitis delta” and “sub-Saharan Africa” and the countries therein for studies published in any language between Jan 1, 1995, and Aug 30, 2016 (appendix). We identified no systematic reviews of hepatitis D virus seroprevalence in the region. In narrative reviews, cited studies predominantly done in west Africa suggested a seroprevalence of between 12% and 67% from diverse populations in Gabon, Cameroon, and Nigeria. We identified no studies in which risk factors for hepatitis D virus infection or a possible association between hepatitis D virus infection and HIV status were analysed.

**Added value of this study**

In our analysis, we systematically compiled available data for hepatitis D virus epidemiology in sub-Saharan Africa, added novel cohort data from Malawi and Ghana, and provided pooled estimates of seroprevalence by region and population group. Our findings suggest substantial geographical variability, with pockets of hyperendemicity in central Africa and low seroprevalence in southern Africa. Overall, hepatitis D virus seroprevalence among HBsAg-positive populations in west and central Africa exceeds the estimated global seroprevalence. Published data do not allow firm conclusions to be drawn about risk factors for hepatitis D virus infection, including any possible association with HIV status. In most studies, detection of anti-hepatitis D virus was not confirmed with a second assay, and hepatitis D virus RNA detection was reported in only a few studies.

**Implications of all the available evidence**

Hepatitis D virus infection makes a potentially important contribution to the burden of liver disease in sub-Saharan Africa, particularly in west and central Africa, but important gaps in knowledge remain and more research is needed. Epidemiological data for east and southern Africa are insufficient. The natural history of infection with genotypes 5–8, which are endemic to Africa, remains to be characterised, including ascertainment of prevalence among patients with well characterised liver disease. Methods of detection of hepatitis D virus RNA need to be standardised, and the reliability of hepatitis D virus antibody and RNA testing in African settings should be investigated. Studies are needed to identify specific risk factors for hepatitis D virus infection, and guide the formulation of prevention and management policies.

## Methods

### Search strategy and selection criteria

We did a systematic review and meta-analysis of studies of the prevalence of hepatitis D virus in sub-Saharan Africa, including added data for anti-hepatitis D virus and hepatitis D virus RNA prevalence from two HIV-positive cohorts in Ghana and Malawi, where scarce data were previously available. AJS searched PubMed, Embase, and Scopus for studies published in any language between Jan 1, 1995, and Aug 30, 2016, in which the prevalence of anti-hepatitis D virus antibody or hepatitis D virus RNA was reported. Search strings included sub-Saharan Africa, the countries therein, and permutations of hepatitis D virus (appendix). MC and AJS independently assessed articles for inclusion; disagreements were resolved by consensus. Hepatitis D virus seroprevalence was defined as reported detection of anti-hepatitis D virus by enzyme immunoassay in HBsAg-positive patients. To be included in our review, hepatitis D virus seroprevalence, patient selection methods, and the geographical and clinical setting had to be reported in the study.^12^ Data for infants or children whose age was not described were excluded to avoid confounding from potential maternal antibody transfer. Populations undergoing assessment in liver disease clinics and those sampled from other populations (defined as general populations) were analysed separately. We contacted study authors for clarification as required. Genotypic data were compiled from studies in which hepatitis D virus RNA was sequenced. We also searched the public sequence databases European Nucleotide Archive and GenBank with the same search strategy used in our initial search (appendix). We excluded genotypic data from studies of immigrants from sub-Saharan Africa who now reside outside the region. Our study was done in accordance with PRISMA recommendations.^13^

### Statistical analysis

AJS extracted seroprevalence data. Duplicate data from the same locations were excluded. Confidence intervals (CIs) were computed by the Wilson method and pooled seroprevalence was calculated with the DerSimonian-Laird random-effects model with Freeman-Tukey double arcsine transformation.^14^, ^15^ We chose a random-effects model a priori because we anticipated heterogeneity arising from variation in study geography and populations. To avoid small sample bias in the random-effects model, we excluded studies in which fewer than ten patients underwent RNA testing, for our calculation of the pooled estimate of hepatitis D virus RNA positivity in patients seropositive for hepatitis D virus. Between-study heterogeneity was assessed with the *I*^2^ statistic. Seroprevalence in patients with liver disease compared with that in those without liver disease were pooled with a DerSimonian-Laird random-effects model.^14^ Meta-regression was done with a residual maximum-likelihood model to examine for sources of heterogeneity related to study location, rural versus urban setting, and the effect of HIV infection by comparison with data from cohorts in which the HIV status of recruited participants was known. We did sensitivity analyses to investigate the effect of population source and of using potentially unrepresentative samples. Risk of bias was independently assessed by AJS and MC with a prevalence critical appraisal tool.^16^ Publication bias was assessed by inspection of a funnel plot and Egger's test.^17^ Analyses were done with metan, metaprop, and metareg packages in Stata (version 14.2).

### Role of the funding source

The study funder had no role in study design; data collection, analysis, or interpretation; or writing of the report. The corresponding author had full access to all the data in the study and had final responsibility for the decision to submit for publication.

## Results

**Figure 1:**
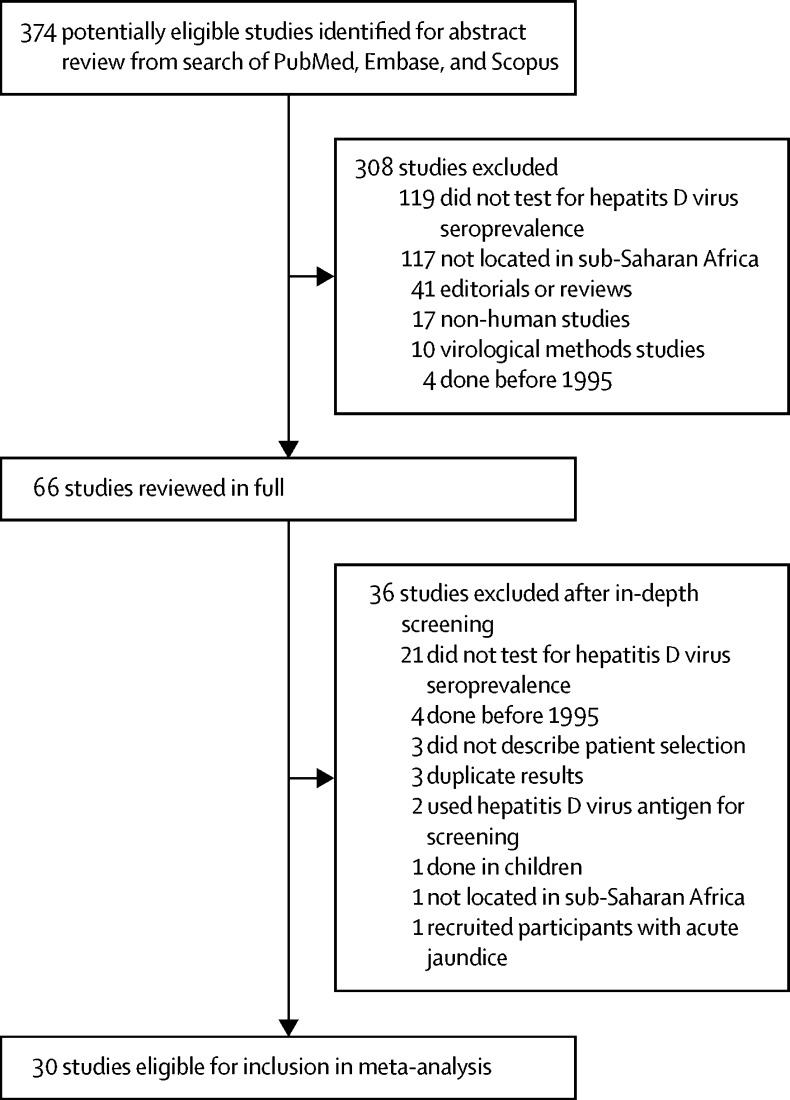
Selection of studies of hepatitis D virus epidemiology in sub-Saharan Africa, 1995–2016, for inclusion in meta-analysis

**Figure 2:**
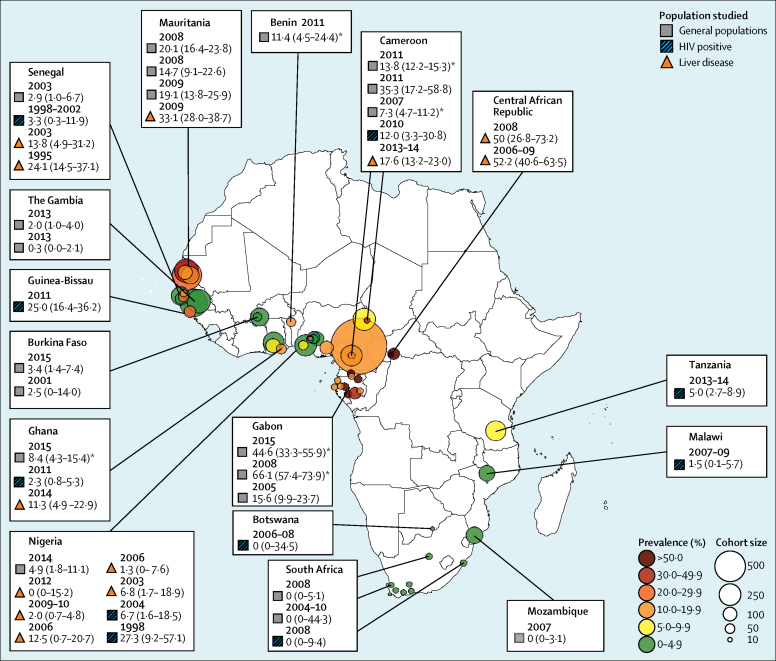
Seroprevalence of hepatitis D virus among HBsAg carriers in sub-Saharan Africa in published studies, 1995–2016 Data in parentheses are 95% CIs. Colour and size of bubble plots show prevalence and cohort size, respectively. *Data obtained in rural settings.

Our search returned 374 records, 30 of which met the inclusion criteria (figure 1). The 30 studies described 40 populations from 15 countries: 23 cohorts from eight west African countries (Benin, Burkina Faso, The Gambia, Ghana, Guinea-Bissau, Mauritania, Nigeria, and Senegal),^18^, ^19^, ^20^, ^21^, ^22^, ^23^, ^24^, ^25^, ^26^, ^27^, ^28^, ^29^, ^30^, ^31^, ^32^, ^33^, ^34^ ten cohorts from three central African countries (Cameroon, Central African Republic, and Gabon),^18^, ^35^, ^36^, ^37^, ^38^, ^39^, ^40^, ^41^, ^42^, ^43^ and seven cohorts from four east or southern African countries (Botswana, Mozambique, South Africa, and Tanzania; figure 2; table).^19^, ^44^, ^45^, ^46^ We added data from previously characterised cohorts co-infected with HIV and hepatitis B virus infection in Malawi and Ghana (appendix).^48^, ^49^ Eight studies included populations^21^, ^33^, ^34^, ^36^, ^37^, ^40^, ^43^, ^46^ from rural settings; the rest were done in urban or mixed populations. Seven commercial enzyme immunoassays were used in studies to test for total anti-hepatitis D virus; in four studies^28^, ^35^, ^36^, ^42^ the test manufacturer was not stated (appendix). Patients were recruited in various settings: through community surveys,^33^, ^34^, ^40^ via blood donation,^25^, ^30^, ^31^, ^34^, ^45^ in antenatal care,^19^, ^21^, ^37^, ^39^, ^44^ during testing of health-care workers,^36^ at HIV clinics,^18^, ^22^, ^23^, ^41^, ^44^, ^46^ and at general medical clinics (patients did not have known liver disease).^25^, ^32^ Eight cohorts comprised exclusively HIV-positive participants.^18^, ^22^, ^23^, ^41^, ^44^, ^46^ HIV testing was done in 13 other cohorts: people with HIV were excluded from seven cohorts,^24^, ^25^, ^30^, ^36^, ^43^, ^44^ whereas the remaining six cohorts^19^, ^21^, ^31^, ^34^, ^37^, ^45^ had a HIV prevalence of 0·6–13·8%. HIV status was not reported in 19 cohorts.^18^, ^20^, ^24^, ^26^, ^27^, ^28^, ^29^, ^32^, ^33^, ^34^, ^35^, ^38^, ^39^, ^40^, ^42^, ^47^ In one study,^31^ HIV prevalence was compared according to hepatitis D virus serostatus, but it lacked statistical power because only six participants seropositive for hepatitis D virus were included.

**Table:** Prevalence of anti-hepatitis D virus and hepatitis D virus RNA in HBsAg-positive general populations and liver disease populations in sub-Saharan Africa, 1995–2015

		**Year**	**Population**	**Tested (n)**	**Anti-hepatitis D virus positive**		Hepatitis D virus RNA positive*	
					n (%)	95% CI	n (%)	95% CI
**General populations**								
West Africa								
	Benin^21^	2011	Pregnant women†	44	5 (11%)	5–24	··	··
	Burkina Faso^31^	2015	Blood donors	177	6 (3%)	1–7	··	··
	Burkina Faso^18^	2001	Mothers	40	1 (3%)	0–14	0 (0)	0–83
	The Gambia^34^	2013	Community members†	394	8 (2%)	1–4	··	··
	The Gambia^34^	2013	Blood donors	292	1 (<1%)	0–2	··	··
	Ghana^33^	2015	Community members†	107	9 (8%)	4–15	··	··
	Ghana‡	2010	Patients from HIV clinics	222	5 (2%)	1–5	2 (40%)	12–77
	Guinea-Bissau^23^	2011	Patients from HIV clinics	72	18 (25%)	16–36	4 (44%)§	19–73
	Mauritania^25^	2008	Blood donors	447	90 (20%)	17–24	56 (62%)	52–72
	Mauritania^26^	2008–09	Pregnant women	109	16 (15%)	9–23	11 (69%)	44–86
	Mauritania^26^	2008–09	Medical outpatients¶	162	31 (19%)	14–26	21 (68%)	50–82
	Nigeria^32^	2014	Medical outpatients¶	103	5 (5%)	2–11	··	··
	Nigeria^18^	2004	Patients from HIV clinics	45	3 (7%)	2–19	2 (67%)	20–94
	Nigeria^18^	1998	Patients from HIV clinics	11	3 (27%)	9–57	0 (0)	0–62
	Senegal^30^	2003	Blood donors	175	5 (3%)	1–7	··	··
	Senegal^22^	1998–2002	Patients from HIV clinics	61	2 (3%)	0–12	··	··
Central Africa								
	Cameroon^42^	2011	National survey participants	1627	225 (14%)	12–15	··	··
	Cameroon^36^	2011	Health-care workers†	17	6 (35%)	17–59	··	··
	Cameroon^41^	2010	Patients from HIV clinics	25	3 (12%)	3–31	1 (33%)	6–80
	Cameroon^37^	2007	Pregnant women†	259	19 (7%)	5–11	··	··
	Gabon^43^	2015	Community members†||	74	33 (45%)	33–56	··	··
	Gabon^39^	2005	Pregnant women	109	17 (16%)	10–24	··	··
	Gabon^40^	2008	Community members†||	124	82 (66%)	57–74	··	··
East or southern Africa								
	Botswana^44^	2006–08	Pregnant women	9	0 (0)	0–35	··	··
	Malawi‡	2007–09	Patients from HIV clinics	133	2 (2%)	0–6	0 (0)	0–71
	Mozambique^45^	2007	Blood donors	146	0 (0)	0–3	··	··
	South Africa^19^	2008	Pregnant women	87	0 (0)	0–5	··	··
	South Africa^44^	2008	HIV-positive pregnant or postnatal women	45	0 (0)	0–9	··	··
	South Africa^44^	2004–10	Pregnant women	6	0 (0)	0–44	··	··
	Tanzania^46^	2013–14	Patients from HIV clinics†	219	11 (5%)	3–9	0 (0)	0–30
**Liver disease populations**								
West Africa								
	Ghana^20^	2014	Patients from hepatology clinics	53	6 (11%)	5–23	··	··
	Mauritania^24^	2009	Patients from hepatology clinics	296	98 (33%)	28–39	61 (62%)	52–71
	Nigeria^28^	2012	Patients with HCC	26	0 (0)	0–15	··	··
	Nigeria^29^	2009–10	Patients from hepatology clinics (15% had cirrhosis, 3% had HCC)	245	5 (2%)	1–5	··	··
	Nigeria^47^	2006	Patients from hepatology clinics (22% had cirrhosis, 51% had HCC)	96	12 (13%)	1–21	··	··
	Nigeria^18^	2006	Patients from hepatology clinics	78	1 (1%)	0–8	1 (100%)	17–100
	Nigeria^18^	2003	Patients from hepatology clinics	44	3 (7%)	2–19	0 (0)	0–62
	Senegal^30^	2003	Patients from hepatology clinics	29	4 (14%)	5–31	··	··
	Senegal^27^	1995	Patients from hepatology clinics (39% had cirrhosis, 57% had HCC)	54	13 (24%)	15–37	··	··
Central Africa								
	Cameroon^38^	2008–09	Patients from hepatology clinics	233	41 (18%)	13–23	25 (61%)	42–74
	Central African Republic^18^	2009	Patients from hepatology clinics	14	7 (50%)	27–73	5 (71%)	35–92
	Central African Republic^35^	2006–09	Patients with HCC	69	36 (52%)	41–64	··	··

HCC=hepatocellular carcinoma.

* Percentages are calculated in the group of patients who are anti-hepatitis D virus positive.

† Study was done in a rural setting.

‡ Data are from the present study.

§ Only nine of 18 samples were tested for hepatitis D virus RNA.

¶ The study population was attendees at a general (non-hepatology) medical clinic without known liver disease.

|| Cluster-sampling community survey.

**Figure 3:**
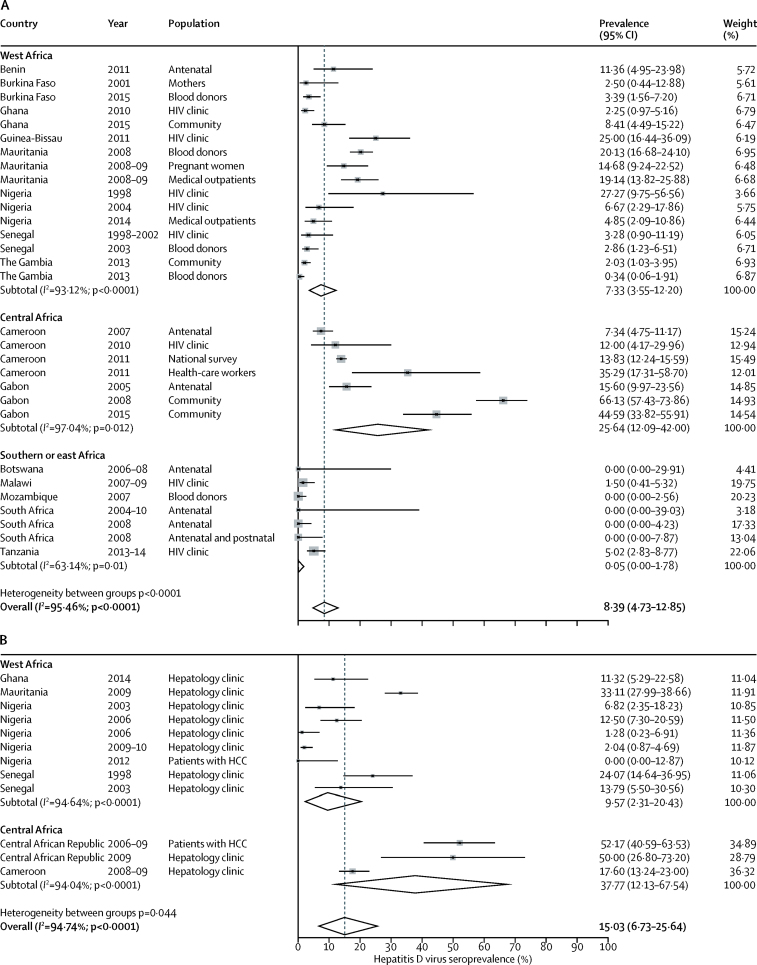
Forest plots of hepatitis D virus seroprevalence among HBsAg-positive patients in sub-Saharan Africa 1995–2016 in general populations (A) and liver-disease populations (B) Data are from a random-effects model. HCC=hepatocellular carcinoma.

Among general populations with HBsAg, hepatitis D virus seroprevalence varied widely within geographical regions, from 0·34% to 27·27% in adults in west Africa,^18^, ^21^, ^25^, ^26^, ^30^, ^31^, ^32^ 7·34% to 66·13% in central Africa,^36^, ^37^, ^39^, ^40^, ^43^ and 0·00% to 5·02% in east and southern Africa^19^, ^44^, ^45^, ^46^ (figure 3A; table). One cluster-sampling survey^40^ done in a rural area in northern Gabon was an extreme outlier, with a seroprevalence of 66·13%. Pooled overall seroprevalence of hepatitis D virus was 8·39% (95% CI 4·73–12·85; figure 3A). Seroprevalence was 7·33% in west Africa (95% CI 3·55-12·20), 25·64% in central Africa (95% CI 12·09–42·00), and 0·05% (95% CI 0·00–1·78%) in east and southern Africa; study region was significantly associated with seroprevalence (p=0·01). According to meta-regression analysis, relative to the reference category of west Africa, seroprevalence was significantly higher in central Africa (coefficient 0·17 [95% CI 0·04–0·30]; p=0·012), but was not significantly different in east or southern Africa (coefficient −0·07 [95% CI −0·21 to 0·07]; p=0·31). The seroprevalence of hepatitis D virus was significantly higher in studies in rural areas than in those in urban areas, but not after adjustment for confounding by African region (coefficient for rural dwelling 0·10 [95% CI −0·03 to 0·24]; p=0·13). Among the 21 studies in which HIV status was reported, seroprevalence of hepatitis D virus did not differ according to HIV prevalence, after adjustment for African region (coefficient per 10% increase in HIV prevalence −0·0002 [95% CI −0·01 to 0·01]; p=0·97).

**Figure 4:**
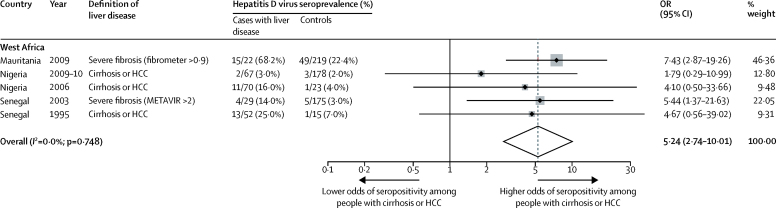
Forest plot of hepatitis D virus seroprevalence among patients with liver disease compared with asymptomatic controls in sub-Saharan Africa Data are from a random-effects model. METAVIR is a histological scoring system based on liver biopsy. OR=odds ratio. HCC=hepatocellular carcinoma.

Among HBsAg-positive patients recruited from hepatology clinics, seroprevalence ranged from 0·00% to 33·11% among nine cohorts in Ghana, Mauritania, Nigeria, and Senegal,^18^, ^20^, ^24^, ^27^, ^28^, ^29^, ^30^, ^47^ with a pooled estimated seroprevalence of 9·57% (95% CI 2·31–20·43) in west Africa (figure 3B). In central Africa, the pooled prevalence from three studies^18^, ^35^, ^38^ from Cameroon and Central African Republic was 37·77% (95% CI 12·13–67·54). We noted significant heterogeneity between west and central Africa (p=0·04; figure 3B; table). In the five studies^24^, ^27^, ^29^, ^30^, ^47^ from Mauritania, Nigeria, and Senegal in which patients with evidence of liver disease (severe fibrosis, cirrhosis, or hepatocellular carcinoma) were directly compared with asymptomatic controls without evidence of liver disease drawn from the same population, the pooled odds ratio of the presence of anti-hepatitis D virus among HBsAg-positive patients with liver disease was 5·24 (95% CI 2·74–10·01; p<0·0001; figure 4).

Testing for hepatitis D virus RNA by qualitative PCR in patients seropositive for hepatitis D virus was done in 324 patients (174 from general populations and 150 from liver-disease populations) in 16 populations.^18^, ^22^, ^23^, ^24^, ^25^, ^26^, ^41^, ^44^, ^46^ RNA positivity ranged from 0% to 69% in general populations, and from 0% to 71% in liver-disease populations (table), but some studies had very small patient numbers. Three studies^25^, ^26^ in blood donors, pregnant women, and medical inpatients from a site in Mauritania were included, in which the prevalence of RNA detected ranged from 62% to 69%, with a pooled estimate of 64% (95% CI 56–73). In a study^46^ done in Tanzania, no hepatitis D virus RNA was detected in 11 participants with HIV who were positive for anti-hepatitis D virus. In two studies in patients with liver disease from Mauritania and Central African Republic, RNA detection rates were 62% and 61% respectively, with a pooled estimate of 62% (95% CI 54–70).^24^, ^38^

**Figure 5:**
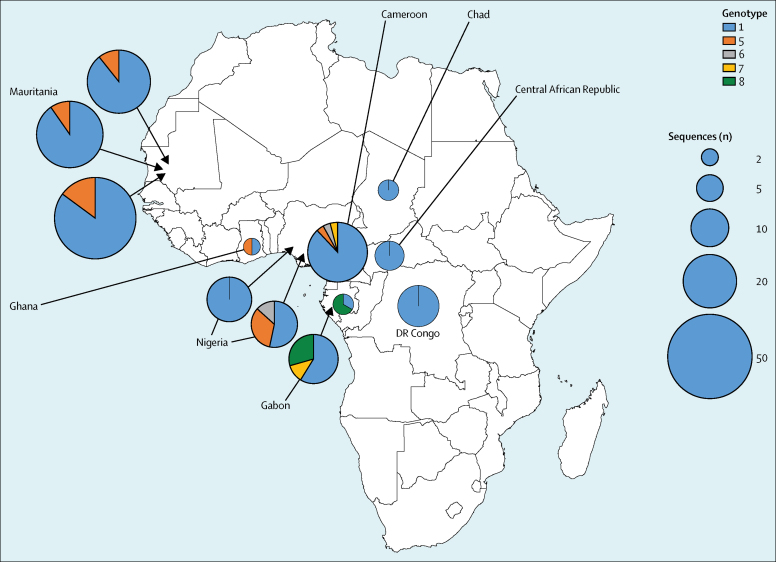
Distribution of hepatitis D virus genotypes in sub-Saharan Africa Genotype data were derived from included studies and publicly available sequences deposited in Genbank and the European Nucleotide Archive database. Each bubble represents an individual study.

Hepatitis D virus genotype data were included in seven studies.^18^, ^24^, ^25^, ^26^, ^38^, ^39^, ^40^ We identified two additional studies by searching public sequence databases. Of the 203 sequences available, genotype 1 was the most prevalent (median 88% [IQR 57–100]; figure 5; appendix).

**Figure 6:**
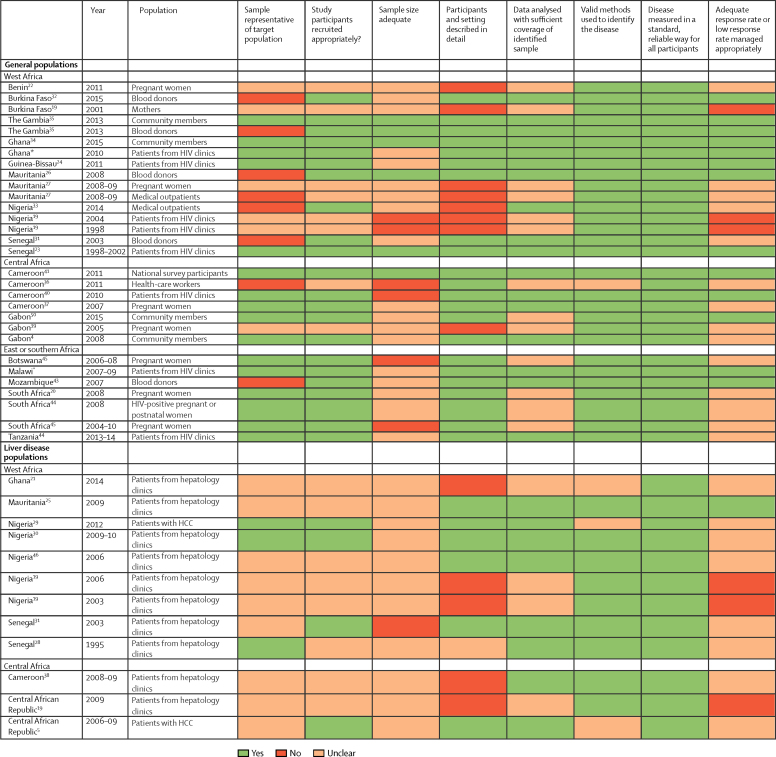
Quality assessment of included studies HCC=hepatocellular carcinoma. *Data are from the present study.

In our quality assessment, we identified recurrent issues with respect to incomplete description of study populations and sampling methods (figure 6). Testing methods did not commonly include confirmation of anti-hepatitis D virus seropositivity by either retesting or hepatitis D virus RNA detection. In most studies, hepatitis D virus epidemiology was not a primary outcome (data not shown), and none of the studies included sample size calculations or estimates of the completeness of the data, including the proportion of eligible people who participated in the study. In several studies, populations were selected that might have not been representative of the general population, including blood donors, health-care workers, and medical outpatients (figure 6). In a sensitivity analysis, exclusion of these studies did not affect estimates of overall prevalence of hepatitis D virus infection (p=0·92). Furthermore, in studies done in the general population, the type of patient recruited or location of recruitment (eg, blood donors, perinatal care, community, health-care workers, medical outpatients, HIV clinics) was not significantly associated with hepatitis D virus seroprevalence (p=0·30). No evidence of publication bias was evident in the funnel plot (appendix) or by the Egger's test (p=0·78).

## Discussion

Epidemiological data for the prevalence of hepatitis D virus are needed to inform drug development and guide the formulation of policy on prevention, diagnosis, and management of the infection in sub-Saharan Africa, in agreement with recommendations from WHO.^51^ In this systematic review and meta-analysis, the estimated prevalence of anti-hepatitis D virus among general HBsAg-positive populations in sub-Saharan Africa was 8·39% (95% CI 4·73–12·85), which exceeded the estimated global prevalence of 5%.^5^ Relating these findings to the prevalence of hepatitis B virus infection would provide an estimated population prevalence of hepatitis B virus and D virus co-infection in sub-Saharan Africa of 0·7%, corresponding to around 7 million people.

Enzyme immunoassay, the method of screening for hepatitis D virus that was used in the included studies, provides case ascertainment of superinfection with hepatitis D virus in carriers of hepatitis B virus, but is thought to be less reliable for the detection of simultaneous hepatitis B virus and D virus co-infection because of high rates of virus clearance and subsequent disappearance of anti-hepatitis D virus.^5^, ^52^ Hepatitis D virus RNA was measured in participants positive for anti-hepatitis D virus in only 16 cohorts, only ten of which tested more than ten participants. In Mauritania, prevalence of RNA detection was consistent across populations, including pregnant women, blood donors, medical outpatients, and attendees at hepatology clinics, at around 62–69%. RNA was detected at a similar frequency in patients with liver disease in central Africa. Available data overall suggest persistent active hepatitis D virus replication in most patients positive for anti-hepatitis D virus, at least in settings with high seroprevalence. In settings with low seroprevalence, RNA was tested for in too few participants to draw firm conclusions about the prevalence of RNA positivity, and the resulting wide CIs overlap with those for estimates of RNA positivity in high-prevalence settings.

Hepatitis D virus seroprevalence was highest among HBsAg-positive populations in central Africa, and lowest in east and southern Africa, although more data are needed from the latter two regions to confirm these findings. Even across similar populations from close geographical regions, seroprevalence was notably heterogeneous. Consistent testing methods (ie, seven commercial assays) were used in all published studies, and thus available data suggest localised clusters of endemicity across sub-Saharan Africa. We did not identify an association between HIV status and hepatitis D virus seroprevalence, although HIV-positive and HIV-negative people in the same population were directly compared in only one study. Furthermore, HIV status was not ascertained in almost half the included studies. Thus, the absence of an association with HIV status should be interpreted with caution, and this finding contrasts with those from the USA and Taiwan, where increasing age, hepatitis C virus co-infection, and intravenous drug use were additonal risk factors for hepatitis D virus seropositivity.^53^, ^54^ Risk factors for hepatitis D virus infection in sub-Saharan Africa have not been established so far. Rural versus urban dwelling was not a risk factor after adjustment for confounding by geographical region in our analysis.

Hepatitis D virus infection is associated with accelerated progression to cirrhosis and increased incidence of hepatocellular carcinoma in carriers of hepatitis B virus, and cross-sectional assessments of hepatitis D virus seroprevalence can therefore be affected by survivorship bias.^5^, ^52^ In European populations, 15% of patients with chronic hepatitis D virus infection progress to cirrhosis within 1–2 years, and 70–80% progress within 5–10 years.^55^ Natural history data have not been reported in African populations or for hepatitis D virus genotypes 5–8, which are specific to the continent. Studies in which convenience sampling of hepatology units was done are helpful to capture the burden of hepatitis D virus among patients with severe liver disease, and our analysis suggests an increased likelihood of seropositivity among HBsAg-positive people with liver disease compared with asymptomatic controls without liver disease in sub-Saharan Africa. However, data are insufficient to derive an estimation of the burden of liver disease attributable to hepatitis D virus in sub-Saharan Africa, because liver disease was incompletely characterised as a result of the poor availability of validated measures of liver fibrosis.^56^

Our analysis has several limitations, which point to important research needs. First, we grouped various populations to facilitate summarising of the data. Published studies were few, addressed populations in various settings, and often had small sample sizes. Convenience sampling often included potentially unrepresentative populations, such as medical outpatients or health-care workers, although exclusion of these studies in sensitivity analyses did not affect estimates of hepatitis D virus seroprevalence in general populations. Substantial resources are needed to provide reliable prevalence estimates—sample sizes are large if drawn from unbiased community sampling, and have to be adjusted by the population prevalence of hepatitis B virus infection. Second, selection and method of ascertainment of the underlying hepatitis B virus infection are likely to affect hepatitis D virus estimates, and were frequently poorly described in the included studies. Third, serological results should ideally be substantiated with measurement of hepatitis D virus RNA to show active replication, but such confirmation was infrequently reported in included studies, raising the possibility of insufficient specificity of seroprevalence data even when commercial assays were used.^57^ RNA testing is not without problems, however. Evidence from an international external quality-control assessment suggested frequent false-negative results among 28 participating laboratories in 17 countries, particularly with the Africa-specific genotypes 5–8; thus, RNA detection might not provide adequate confirmation without rigorous standardisation.^58^ Finally, our regional pooled estimates of hepatitis D virus seroprevalence have wide CIs, reflecting the substantial heterogeneity of the available data.^59^ We adopted a random-effects model a priori to account for anticipated heterogeneity.^12^

In summary, our analysis provides a preliminary estimate of the epidemiology of hepatitis D virus infection in sub-Saharan Africa, and points to several important gaps in knowledge and directions for future research. Results suggest that co-infection with hepatitis B and D viruses could represent an important cause of liver disease in some regions and populations. Improved and systematically collected epidemiology data are needed, particularly for east and southern Africa, as is identification of risk factors for hepatitis D virus transmission. A clinical characterisation of hepatitis D virus infection is needed, particularly for the genotypes 5–8. A focus on patients with established liver disease is required to ascertain the burden of adverse hepatic outcomes attributable to hepatitis D virus. Future research initiatives should be accompanied by an assessment of the reliability of testing methods in the African setting.

Hepatitis B virus infection remains endemic across sub-Saharan Africa^60^ and improved implementation of measures to prevent hepatitis B virus infection—including vaccination, prevention of needle reuse in health care, quality-assured transfusion screening, and expansion of diagnosis and treatment services—is required to reduce the burden of both infections.^50^ Mathematical modelling suggests that maintaining infant hepatitis B virus vaccination coverage above 80% and improving birth-dose vaccination coverage for children born to HBsAg-positive mothers (presently less than 10% in sub-Saharan Africa), represents an effective strategy for the eradication of both hepatitis B and D virus infection.^61^, ^62^

## References

[1] Global Burden of Disease Study 2013 Collaborators (2015). Global, regional, and national incidence, prevalence, and years lived with disability for 301 acute and chronic diseases and injuries in 188 countries, 1990–2013: a systematic analysis for the Global Burden of Disease Study 2013. Lancet.

[2] Schweitzer A, Horn J, Mikolajczyk RT, Krause G, Ott JJ (2015). Estimations of worldwide prevalence of chronic hepatitis B virus infection: a systematic review of data published between 1965 and 2013. Lancet.

[3] Hughes SA, Wedemeyer H, Harrison PM (2011). Hepatitis delta virus. Lancet.

[4] Rizzetto M (2015). Hepatitis D virus: introduction and epidemiology. Cold Spring Harb Perspect Med.

[5] Lempp FA, Ni Y, Urban S (2016). Hepatitis delta virus: insights into a peculiar pathogen and novel treatment options. Nat Rev Gastroenterol Hepatol.

[6] Matthews PC, Geretti AM, Goulder PJ, Klenerman P (2014). Epidemiology and impact of HIV coinfection with hepatitis B and hepatitis C viruses in Sub-Saharan Africa. J Clin Virol.

[7] UNAIDS (2014). HIV/AIDS estimates: core epidemiological slides. http://www.unaids.org/sites/default/files/media_asset/20150714_epi_core_en.pdf.

[8] Lacombe K, Rockstroh J (2012). HIV and viral hepatitis coinfections: advances and challenges. Gut.

[9] Fernandez-Montero JV, Vispo E, Barreiro P (2014). Hepatitis delta is a major determinant of liver decompensation events and death in hiv-infected patients. Clin Infect Dis.

[10] Koh C, Canini L, Dahari H (2015). Oral prenylation inhibition with lonafarnib in chronic hepatitis D infection: a proof-of-concept randomised, double-blind, placebo-controlled phase 2A trial. Lancet Infect Dis.

[11] Bogomolov P, Alexandrov A, Voronkova N (2016). Treatment of chronic hepatitis D with the entry inhibitor myrcludex B: First results of a phase Ib/IIa study. J Hepatol.

[12] Higgins JPT, Thompson SG, Spiegelhalter DJ (2009). A re-evaluation of random-effects meta-analysis. J R Stat Soc Ser A.

[13] Moher D, Liberati A, Tetzlaff J, Altman DG (2009). Preferred reporting items for systematic reviews and meta-analyses: the PRISMA statement. BMJ.

[14] DerSimonian R, Laird N (1986). Meta-analysis in clinical trials. Control Clin Trials.

[15] Freeman MF, Tukey JW (1950). Transformations related to the angular and the square root. Ann Math Stat.

[16] Munn Z, Moola S, Lisy K, Riitano D, Tufanaru C (2015). Methodological guidance for systematic reviews of observational epidemiological studies reporting prevalence and cumulative incidence data. Int J Evid Based Healthc.

[17] Egger M, Davey Smith G, Schneider M, Minder C (1997). Bias in meta-analysis detected by a simple, graphical test. BMJ.

[18] Andernach IE, Leiss LV, Tarnagda ZS (2014). Characterization of hepatitis delta virus in sub-Saharan Africa. J Clin Microbiol.

[19] Andersson MI, Maponga TG, Ijaz S (2013). The epidemiology of hepatitis B virus infection in HIV-infected and HIV-uninfected pregnant women in the Western Cape, South Africa. Vaccine.

[20] Asmah RH, Boamah I, Afodzinu M (2014). Prevalence of hepatitis D infection in patients with hepatitis B virus-related liver diseases in Accra, Ghana. West Afr J Med.

[21] De Paschale M, Ceriani C, Cerulli T (2014). Prevalence of HBV, HDV, HCV, and HIV infection during pregnancy in northern Benin. J Med Virol.

[22] Diop-Ndiaye H, Toure-Kane C, Etard JF (2008). Hepatitis B, C seroprevalence and delta viruses in HIV-1 Senegalese patients at HAART initiation (retrospective study). J Med Virol.

[23] Langhoff Hønge B, Jespersen S, Medina C (2014). Hepatitis B and delta virus are prevalent but often subclinical co-infections among HIV infected patients in Guinea-Bissau, West Africa: a cross-sectional study. PLoS One.

[24] Lunel-Fabiani F, Mansour W, Amar AO (2013). Impact of hepatitis B and delta virus co-infection on liver disease in Mauritania: a cross sectional study. J Infect.

[25] Mansour W, Bollahi MA, Hamed CT (2012). Virological and epidemiological features of hepatitis delta infection among blood donors in Nouakchott, Mauritania. J Clin Virol.

[26] Mansour W, Malick FZ, Sidiya A (2012). Prevalence, risk factors, and molecular epidemiology of hepatitis B and hepatitis delta virus in pregnant women and in patients in Mauritania. J Med Virol.

[27] Mbaye PS, Renaudineau Y, Diallo A (2000). Hepatitis C virus and chronic hepatopathies in Dakar: case-control study. Med Trop (Mars).

[28] Olal SO, Akere A, Otegbayo JA (2012). Are patients with primary hepatocellular carcinoma infectious of hepatitis B, C and D viruses?. Afr J Med Med Sci.

[29] Onyekwere CA, Audu RA, Duro-Emmanuel F, Ige FA (2012). Hepatitis D infection in Nigeria. Indian J Gastroenterol.

[30] Vray M, Debonne JM, Sire JM (2006). Molecular epidemiology of hepatitis B virus in Dakar, Sénégal. J Med Virol.

[31] Sawadogo A, Ouédraogo AS, Poda A (2016). Seroprevalence of hepatitis D virus among blood donors with antigene HBs at Bobo-Dioulasso regional transfusion center, Burkina Faso. J Afr Hepato Gastroenterol.

[32] Opaleye OO, Japhet OM, Adewumi OM (2016). Molecular epidemiology of hepatitis D virus circulating in Southwestern Nigeria. Virol J.

[33] Ampah KA, Pinho-Nascimento CA, Kerber S (2016). Limited genetic diversity of hepatitis B virus in the general population of the Offin River Valley in Ghana. PLoS One.

[34] Lemoine M, Shimakawa Y, Njie R, on behalf of the PROLIFICA investigators (2016). Acceptability and feasibility of a screen-and-treat programme for hepatitis B virus infection in The Gambia: the Prevention of Liver Fibrosis and Cancer in Africa (PROLIFICA) study. Lancet Glob Health.

[35] Bekondi C, Mobima T, Ouavene JO (2010). Etiopathological factors of hepatocellular carcinoma in Bangui, Central African Republic: clinical, biological characteristics and virological aspects of patients. Pathol Biol (Paris).

[36] Birguel J, Ndong JG, Akhavan S (2011). Viral markers of hepatitis B, C and D and HB vaccination status of a health care team in a rural district of Cameroon. Med Trop (Mars).

[37] Ducancelle A, Abgueguen P, Birguel J (2013). High endemicity and low molecular diversity of hepatitis B virus infections in pregnant women in a rural district of North Cameroon. PLoS One.

[38] Foupouapouognigni Y, Noah DN, Sartre MT, Njouom R (2011). High prevalence and predominance of hepatitis delta virus genotype 1 infection in Cameroon. J Clin Microbiol.

[39] Makuwa M, Caron M, Souquiere S, Malonga-Mouelet G, Mahe A, Kazanji M (2008). Prevalence and genetic diversity of hepatitis B and delta viruses in pregnant women in Gabon: molecular evidence that hepatitis delta virus clade 8 originates from and is endemic in central Africa. J Clin Microbiol.

[40] Makuwa M, Mintsa-Ndong A, Souquiere S, Nkoghe D, Leroy EM, Kazanji M (2009). Prevalence and molecular diversity of hepatitis B virus and hepatitis delta virus in urban and rural populations in northern Gabon in central Africa. J Clin Microbiol.

[41] Salpini R, Fokam J, Ceccarelli L (2016). High burden of HBV-infection and atypical HBV strains among HIV-infected Cameroonians. Curr HIV Res.

[42] Njouom R, Tejiokem MC, Texier G, Fontanet A (2015). Prevalence of hepatitis B, hepatitis C and hepatitis D virus infections in Cameroon: results from a national population based survey (the ANRS 12289 project). J Viral Hepat.

[43] Francois-Souquiere S, Makuwa M, Bisvigou U, Kazanji M (2016). Epidemiological and molecular features of hepatitis B and hepatitis delta virus transmission in a remote rural community in central Africa. Infect Genet Evol.

[44] Matthews PC, Beloukas A, Malik A (2015). Prevalence and characteristics of hepatitis B virus (HBV) coinfection among HIV-positive women in South Africa and Botswana. PLoS One.

[45] Cunha L, Plouzeau C, Ingrand P (2007). Use of replacement blood donors to study the epidemiology of major blood-borne viruses in the general population of Maputo, Mozambique. J Med Virol.

[46] Winter A, Letang E, Vedastus Kalinjuma A (2016). Absence of hepatitis delta infection in a large rural HIV cohort in Tanzania. Int J Infect Dis.

[47] Nwokediuko SC, Ijeoma U (2009). Seroprevalence of antibody to HDV in Nigerians with hepatitis B virus-related liver diseases. Niger J Clin Pract.

[48] Aoudjane S, Chaponda M, Gonzalez Del Castillo AA (2014). Hepatitis B virus sub-genotype A1 infection is characterized by high replication levels and rapid emergence of drug resistance in HIV-positive adults receiving first-line antiretroviral therapy in Malawi. Clin Infect Dis.

[49] Stockdale AJ, Phillips RO, Beloukas A (2015). Liver fibrosis by transient elastography and virologic outcomes after introduction of tenofovir in lamivudine-experienced adults with HIV and hepatitis B virus coinfection in Ghana. Clin Infect Dis.

[50] Lemoine M, Thursz MR (2017). Battlefield against hepatitis B infection and HCC in Africa. J Hepatol.

[51] WHO (2016). Combating hepatitis B and C to reach elimination by 2030.

[52] Alfaiate D, Dény P, Durantel D (2015). Hepatitis delta virus: From biological and medical aspects to current and investigational therapeutic options. Antiviral Research.

[53] Kushner T, Serper M, Kaplan DE (2015). Delta hepatitis within the Veterans Affairs medical system in the United States: prevalence, risk factors, and outcomes. J Hepatol.

[54] Lin HH, Lee SS, Yu ML (2015). Changing hepatitis D virus epidemiology in a hepatitis B virus endemic area with a national vaccination program. Hepatology.

[55] Sureau C, Negro F (2016). The hepatitis delta virus: replication and pathogenesis. J Hepatol.

[56] Lemoine M, Eholie S, Lacombe K (2015). Reducing the neglected burden of viral hepatitis in Africa: strategies for a global approach. J Hepatol.

[57] King S, Adjei-Asante K, Appiah L (2015). Antibody screening tests variably overestimate the prevalence of hepatitis C virus infection among HIV-infected adults in Ghana. J Viral Hepat.

[58] Le Gal F, Brichler S, Sahli R, Chevret S, Gordien E (2016). First international external quality assessment for hepatitis delta virus RNA quantification in plasma. Hepatology.

[59] Greenland S (1994). Can meta-analysis be salvaged?. Am J Epidemiol.

[60] Lozano R, Naghavi M, Foreman K (2012). Global and regional mortality from 235 causes of death for 20 age groups in 1990 and 2010: a systematic analysis for the Global Burden of Disease Study 2010. Lancet.

[61] Goyal A, Murray JM (2014). The impact of vaccination and antiviral therapy on hepatitis B and hepatitis D epidemiology. PLoS One.

[62] Subaiya S, Dumolard L, Lydon P, Gacic-Dobo M, Eggers R, Conklin L (2015). Global routine vaccination coverage, 2014. MMWR Morb Mortal Wkly Rep.

