# Correction to *Lancet Glob Health* 2016; 5: e992–1003

**DOI:** 10.1016/S2214-109X(17)30381-9

**Published:** 2017-10-09

*Stockdale AJ, Chaponda M, Beloukas A, et al. Prevalence of hepatitis D virus infection in sub-Saharan Africa: a systematic review and meta-analysis.* Lancet Glob Health *2017; **5:** e992–e1003*—The appendix of this Article should have contained a reference list. The appendix has been corrected as of Oct 9, 2017.

