# Supplementary material for: Prevalence of hepatitis D virus infection in sub-Saharan Africa: a systematic review and meta-analysis
Source: Lancet Glob Health. 2017 Sep 11;5(10):e992–e1003. doi: 10.1016/S2214-109X(17)30298-X (PMC5599428; doi:10.1016/S2214-109X(17)30298-X)

THE LANCET
Global Health

Supplementary appendix

This appendix formed part of the original submission and has been peer reviewed. We post it as supplied by the authors.

Supplement to: Stockdale AJ, Chaponda M, Beloukas A, et al. Prevalence of hepatitis D virus infection in sub-Saharan Africa: a systematic review and meta-analysis. *Lancet Glob Health* 2017; **5:** e992–1003.

SUPPLEMENTARY MATERIAL

Supplementary Table 1: Search strings performed using Pubmed, Embase and Scopus

Limits: publication date 1 Jan 1995- 1 June 2016
No language restriction

#1	Hepatitis D[MeSH] OR Hepatitis Delta Virus [MeSH] or Hepatitis delta Antigens [MeSH] or “hepatitis D virus” OR “hepatitis d” or “hepatitis delta” OR hepatitis delta antigen* OR “hepatitis delta virus” or “HDV” or “delta virus”
#2	Search Africa[MeSH] OR africa[tiab] OR "sub Saharan africa"[tiab] OR "sub-Saharan Africa"[tiab] OR Angola[tiab] OR Benin[tiab] OR Botswana[tiab] OR "Burkina Faso"[tiab] OR Burundi[tiab] OR Cameroon[tiab] OR "Cape Verde"[tiab] OR "Central African Republic"[tiab] OR Chad[tiab] OR Comoros[tiab] OR "Republic of the Congo"[tiab] OR "Democratic Republic of the Congo"[tiab] OR "Cote d'Ivoire"[tiab] OR Djibouti[tiab] OR "Equatorial Guinea"[tiab] OR Eritrea[tiab] OR Ethiopia[tiab] OR Gabon[tiab] OR "The Gambia"[tiab] OR Ghana[tiab] OR Guinea[tiab] OR "Guinea-Bissau"[tiab] OR Kenya[tiab] OR Lesotho[tiab] OR Liberia[tiab] OR Madagascar[tiab] OR Malawi[tiab] OR Mali[tiab] OR Mauritania[tiab] OR Mauritius[tiab] OR Mozambique[tiab] OR Namibia[tiab] OR Niger[tiab] OR Nigeria[tiab] OR Rwanda[tiab] OR "Sao Tome and Principe"[tiab] OR Senegal[tiab] OR Seychelles[tiab] OR "Sierra Leone"[tiab] OR "South Africa"[tiab] OR "South Sudan"[tiab] OR Sudan[tiab] OR Swaziland[tiab] OR Tanzania[tiab] OR Togo[tiab] OR Uganda[tiab] OR Zambia[tiab] OR Zimbabwe[tiab]
#3	#1 AND #2

*For Embase, the thesaurus tool was used to map to subject headings.
Abbreviations: [MeSH]= Medical subject heading (for MEDLINE searches), [TIAB]=Title, abstract

Supplementary Table 2. **Prevalence of hepatitis B virus (HBV) and hepatitis D virus (HDV) co-infection in adults attending for routine HIV care in Kumasi, Ghana and Blantyre, Malawi**

	Kumasi, Ghana	Blantyre Malawi
Year of sampling	2010-2013	2007-2009
Tested for HBsAg, n	1643	1117
HBsAg positive, n (%)	230 (14·0)	133 (11·9)
Female, n (%)	152 (66·1)	67 (50·4)
Age, median years (IQR)	39 (33, 46)	36 (31, 42)
HIV-1 RNA, median log_{10} cps/ml (IQR)*	UD (UD, 3·8)	4·0 (3·5, 4·7)
CD4 count, median cells/mm^3 (IQR)	476 (283, 638)	109 (53, 192)
Tested for anti-HDV, n (%)	222 (96·5)	133 (100)
Anti-HDV positive, n (%)	5 (2·2)	2 (1·5)
HDV RNA positive, n (%)[b]	2 (0·9)	0 (0)

*A total of 185/230 (80·4%) HBsAg-positive patients in Kumasi were established on antiretroviral therapy; all patients from Malawi were treatment-naïve at the time of sampling; [b]HDV RNA was detected in two of the seven anti-HDV positive samples, with a mean HDV RNA load from two experiments of 6·0 and 5·0 log_{10} IU/ml (inter-assay coefficient of variation 0·1% and 0·5%, respectively). Abbreviations: HBsAg, hepatitis B surface antigen; IQR, interquartile range; UD, undetectable (<40 copies/ml).

Supplementary Table 3: Method of enzyme immunoassay for total anti-HDV antibody used in included studies

Country	Ref	Year	Population	Method and manufacturer
**General Populations**				
Benin	21	2011	Pregnant women †	ETI-AB-DELTAK-2 (anti-HD), Diasorin, Saluggia, Italy
Burkina Faso	31	2015	Blood donors	ETI-AB-DELTAK-2 (anti-HD), Diasorin
Burkina Faso	18	2001	Mothers	ETI-AB-DELTAK-2 (anti-HD), Diasorin or Murex Anti-Delta, Abbott, IL, USA
The Gambia	34	2013	Community	ETI-AB-DELTAK-2 (anti-HD), Diasorin
The Gambia	34	2013	Blood donors	ETI-AB-DELTAK-2 (anti-HD), Diasorin
Ghana	33	2015	Community †	ETI-AB-DELTAK-2 (anti-HD), Diasorin
Ghana	S+	2010	HIV clinic	HDV Ab, Dia.Pro, Milan, Italy
Guinea-Bissau	23	2011	HIV clinic	ETI-AB-DELTAK-2 (anti-HD), Diasorin
Mauritania	25	2008	Blood donors	ETI-AB-DELTAK-2 (anti-HD), Diasorin
Mauritania	26	2008-9	Pregnant women	ETI-AB-DELTAK-2 (anti-HD), Diasorin
Mauritania	26	2008-9	Medical outpatients ‡	ETI-AB-DELTAK-2 (anti-HD), Diasorin
Nigeria	32	2014	Medical outpatients ‡	ETI-AB-DELTAK-2 (anti-HD), Diasorin
Nigeria	18	2004	HIV clinic	ETI-AB-DELTAK-2 (anti-HD), Diasorin or Murex Anti-Delta, Abbott
Nigeria	18	1998	HIV clinic	ETI-AB-DELTAK-2 (anti-HD), Diasorin or Murex Anti-Delta, Abbott
Senegal	30	2003	Blood donors	
Senegal	22	1998-02	HIV clinic	Murex Anti-Delta, Abbott
Cameroon	42	2011	National survey	Not stated
Cameroon	36	2011	Health care workers †	Not stated
Cameroon	41	2010	HIV clinic	Anti-HDV, Radim Alisei, Pomezia, Italy
Cameroon	37	2007	Pregnant women †	ETI-AB-DELTAK-2 (anti-HD), Diasorin
Gabon	64	2015	Community†§	Murex Anti-Delta, Abbott,
Gabon	39	2005	Pregnant women	Murex Anti-Delta, Abbott,
Gabon	40	2008	Community †§	Murex Anti-Delta, Abbott,
Botswana	45	2006-8	Pregnant women	HDV Ab, Dia.Pro
Malawi	S+	2007-9	HIV clinic	HDV Ab, Dia.Pro
Mozambique	44	2007	Blood donors	ETI-AB-DELTAK-2 (anti-HD), Diasorin
South Africa	19	2008	Pregnant women	ETI-AB-DELTAK-2 (anti-HD), Diasorin
South Africa	45	2008	HIV+ pregnant or postnatal women	HDV Ab, Dia.Pro
South Africa	45	2004-10	Pregnant women	HDV Ab, Dia.Pro
Tanzania	46	2013-14	HIV clinic†	ETI-AB-DELTAK-2 (anti-HD), Diasorin
**Liver disease populations**		Liver disease populations		
Ghana	20	2014	Hepatology clinic	Anti-HDV, Globe Diagnostics, Milan, Italy
Mauritania	24	2009	Hepatology clinic	ETI-AB-DELTAK-2 (anti-HD), Diasorin
Nigeria	28	2012	Patients with HCC	Not stated
Nigeria	29	2009-10	Hepatology clinic (15% cirrhosis, 3% HCC)	HDV Ab, Dia.Pro
Nigeria	49	2006	Hepatology clinic (22% cirrhosis, 51% HCC)	ETI-AB-DELTAK-2 (anti-HD), Diasorin
Nigeria	18	2006	Hepatology clinic	ETI-AB-DELTAK-2 (anti-HD), Diasorin or Murex Anti-Delta, Abbott
Nigeria	18	2003	Hepatology clinic	ETI-AB-DELTAK-2 (anti-HD), Diasorin or Murex Anti-Delta, Abbott
Senegal	30	2003	Hepatology clinic	
Senegal	27	1995	Hepatology clinic (39% cirrhosis, 57% HCC)	Anti-Delta, Sanofi Pasteur, Lyon, France
Cameroon	38	2008-9	Hepatology clinic	Murex Anti-Delta, Abbott
CAR	18	2009	Hepatology clinic	ETI-AB-DELTAK-2 (anti-HD), Diasorin
CAR	35	2006-9	Patients with HCC	Not stated

Supplementary table 4: Distribution of HDV genotypes in sub-Saharan Africa

Study	Year	Genbank/ ENA reference	Country	Sequences	Genotype	Frequency, n(%)
Andernach[18]	2004-6		Nigeria	15	1	8 (53)
					5	5 (33)
		JX888098-JX888135			6	2 (13)
Andernach[18]	2009		CAR	6	1	6 (100)
Andernach[18]	2007		Chad	3	1	3 (100)
Mansour[25]	2008-9	-	Mauritania	28	1	25 (89)
					5	3 (11)
Mansour[26]	2008-9	-	Mauritania	31	1	28 (90)
					5	3 (10)
Lunel-Fabiani[24]	2009	-	Mauritania	47	1	40 (85)
					5	7 (15)
Foupouapouognigni[38]	2008-9	HQ013330-HQ013354	Cameroon	25	1	22 (88)
					5	1 (4)
					6	1 (4)
					7	1 (4)
Makuwa[40]	2005	FJ349279 - FJ349295	Gabon	17	1	10 (59)
					7	2 (12)
					8	5 (29)
Makuwa[39]	2005	EU035518 - EU035520	Gabon	3	1	1 (33)
					8	2 (67)
This study	2015	KU307191 - KU307192	Ghana	2	1	1 (50)
					5	1 (50)
Opaleye[50]	2016	KU844264 - KU844277	Nigeria	14	1	14 (100)
Makiala-Mandanda[65]	2017	LT703299-LT703311	DR Congo	12	1	12 (100)

[a]Nucleotide positions (nt) are relative to NCBI reference sequence NC_001653.2 – the HDV antigen coding sequence is located at nucleotide positions 1013-1600. Data are from included studies that reported genotypic data and from data published in publicly available sequence databases (GenBank and European Nucleotide Archive, available at https://www.ncbi.nlm.nih.gov/genbank/ and http://www.ebi.ac.uk/ena respectively).

Supplementary Material: HDV RNA detection and sequencing in Ghana and Malawi

Methodology

Plasma samples (250µl) underwent nucleic acid extraction by the Nuclisens easyMAG platform (Biomeriuex, Marcy l'Etoile, France) using a modified version of the off-board lysis protocol (B protocol) and 40ul elution volume. The extract (10µl in 25µl final reaction volume) underwent single-step reverse transcription and amplification on the ABI 7500 real-time PCR instrument using the AgPath-ID One-Step kit (Applied Biosystems, Paisley, UK). Primers, probes and PCR conditions were previously published.[66] To produce a quantification standard, a synthetic HDV cDNA plasmid was prepared covering a 53 nucleotide sequence located upstream of the large-HDAg open reading frame. The HDV plasmid was calibrated in triplicate serial dilutions run alongside duplicate serial dilutions of the 1st World Health Organisation (WHO) International Standard for HDV RNA (NIBSC, Paul-Ehrlich-Institute code 7657/12). The assay lower limit of quantification was determined as 144 IU/mL. A HDV RNA positive plasma sample (6.7 $\log_{10}$ IU/mL) was included as a positive control in each set of experiments; with this sample, the inter-assay coefficient of variation (CV) from eight independent experiments was 0.2%.

HDV RNA positive samples underwent Sanger sequencing of a 257-base pair region of large-HDAg open reading frame. Nucleic acid extracts were reverse transcribed by the SuperScript® III One-Step RT-PCR System with Platinum® Taq DNA Polymerase (Invitrogen, MA, USA) under the following thermal cycling conditions: 55 °C for 30 min, 94 °C for 2 min, and then 40 cycles at 94 °C for 15 s, 50 °C for 30 s, 68 °C for 1 min and 68 °C for 5 min. This was followed by nested amplification using Platinum® PCR SuperMix High Fidelity (Invitrogen, CA, USA) under the following conditions: 94 °C for 2 min, and then 35 cycles of 94 °C for 30 s, 54 °C for 30 s, 68 °C for 1 min and 68 °C for 10 min. Nested PCR products were visualised on a 2% agarose gel, purified using the QIAquick PCR purification kit (Qiagen, Hilden, Germany), and sequenced using BigDye Terminator Cycle Sequencing Kit v3.1 on the ABI Prism 3730 Genetic Analyser (Applied Biosystems). Raw sequences were edited using SeqScape version 2.7 (Applied Biosystems), aligned using the ClustalW as implemented in MEGA v.6.0, and manually edited. HDV genotypes were identified phylogenetically using 67 reference sequences of known genotype derived from the NCBI Genbank Database (www.ncbi.nlm.nih.gov/genbank). Phylogenetic analysis was performed based on Bayesian Markov Chain Monte-Carlo (MCMC) phylogenetic inference, as implemented in BEAST v1.8.1 for 10^7 generations, and tree visualization and annotation was performed using FigTree v1.4. Sequences obtained in this study were submitted to NCBI GenBank (accession numbers KU307191-KU307192).

Results

Within our previous studies[47,48], 1643 Ghanaian and 1117 Malawian HIV positive adults were tested for HBsAg (Supplementary Table 2). HBsAg prevalence was 14·0% (95% CI 12·4-15·8) in Ghana and 11·9% (95% CI 10·1-13·9) in Malawi. HDV seroprevalence among HBsAg positive patients was 5/222 (2·2%; 95% CI 0·8-5·3) in Ghana and 2/133 (1·5%; 95% CI 0·1-5·7) in Malawi. HDV RNA was detected in 2/5 and 0/2 anti-HDV positive patients, yielding a HDV RNA prevalence among HBsAg carriers of 0·9% (95% CI 0·03-3·4) and 0% (95% CI 0-3·4), respectively. The two HDV strains from Ghana were genotyped as 1 and 5 (Supplementary Figure 1).

Supplementary Figure 1. Phylogenetic analysis of two HDV strains from Ghana alongside reference sequences of known HDV genotype. One HDV strain clustered within HDV genotype 5 sequences (in red), whereas the second was closely related to HDV genotype 1 sequences (in blue). Nodes with posterior probability equal to 1.0 are indicated with an asterisk.

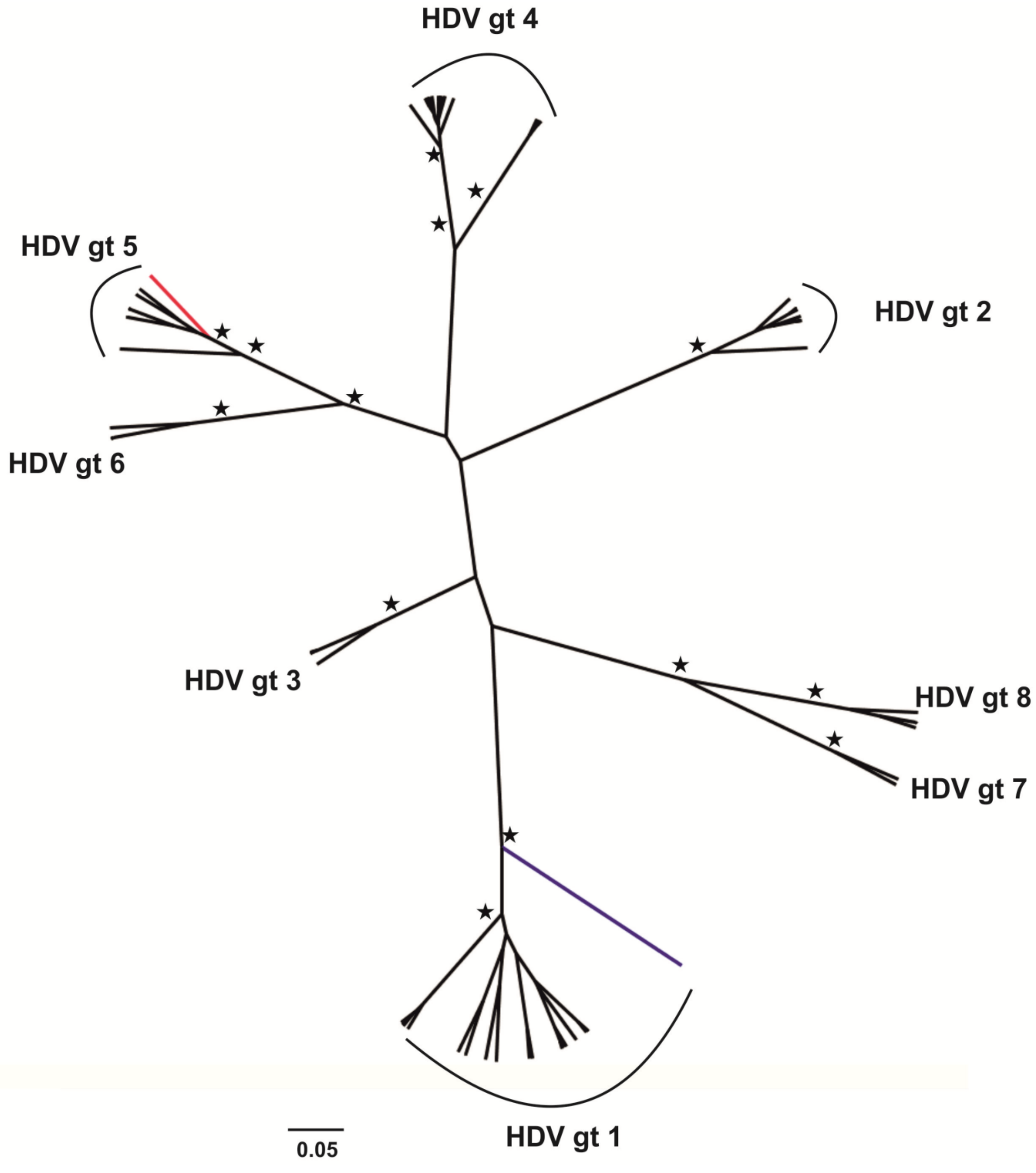

Supplementary Figure 2. Funnel plot of included studies

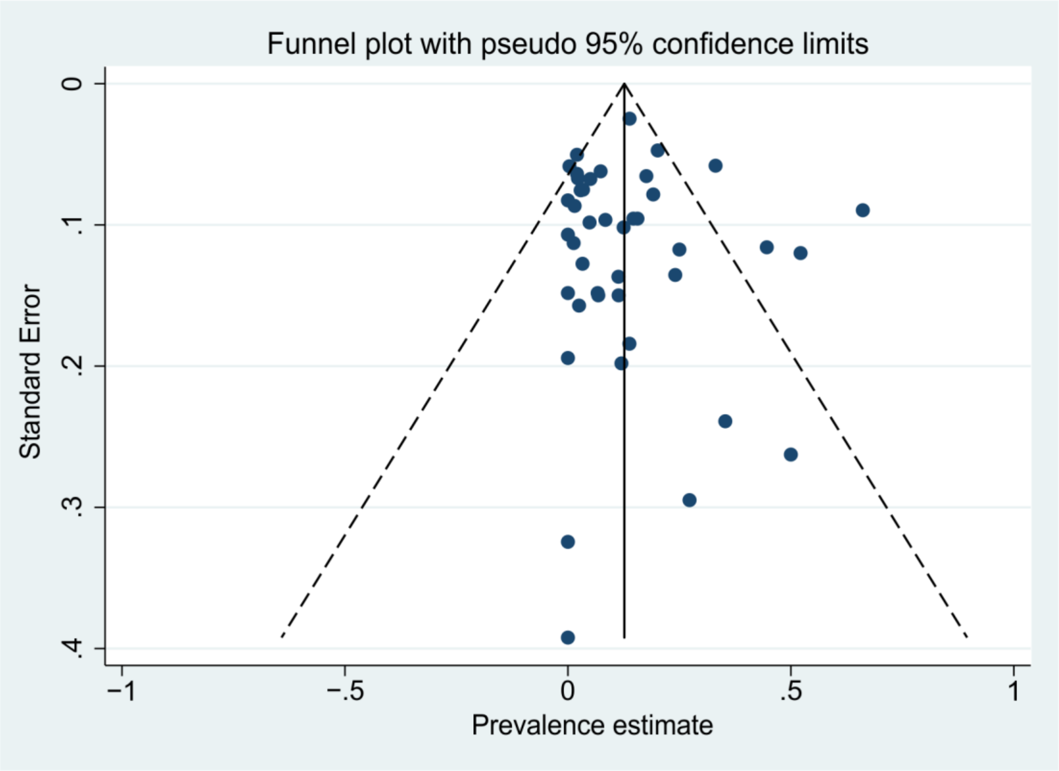

Supplement: Supplementary appendix [file mmc1.pdf]
